# Molecular biomarkers for assessing the heat-adapted phenotype: a narrative scoping review

**DOI:** 10.1186/s12576-023-00882-4

**Published:** 2023-10-17

**Authors:** J. Corbett, J. S. Young, M. J. Tipton, J. T. Costello, T. B. Williams, E. F. Walker, B. J. Lee, C. E. Stevens

**Affiliations:** 1 Extreme Environments Laboratory, School of Sport Health and Exercise Sciences, University of Portsmouth, Portsmouth, UK; 2 National Horizons Centre, Teesside University, Darlington, UK; 3 Defence Science and Technology Laboratory, Porton Down, Salisbury, UK; 4 Occupational and Environmental Physiology Group, Centre for Sport, Exercise and Life Sciences, Faculty of Health and Life Sciences, Coventry University, Coventry, UK

**Keywords:** Heat, Acclimation, Acclimatisation, Hormone, Heat shock protein

## Abstract

Heat acclimation/acclimatisation (HA) mitigates heat-related decrements in physical capacity and heat-illness risk and is a widely advocated countermeasure for individuals operating in hot environments. The efficacy of HA is typically quantified by assessing the thermo-physiological responses to a standard heat acclimation state test (i.e. physiological biomarkers), but this can be logistically challenging, time consuming, and expensive. A valid molecular biomarker of HA would enable evaluation of the heat-adapted state through the sampling and assessment of a biological medium. This narrative review examines candidate molecular biomarkers of HA, highlighting the poor sensitivity and specificity of these candidates and identifying the current lack of a single ‘standout’ biomarker. It concludes by considering the potential of multivariable approaches that provide information about a range of physiological systems, identifying a number of challenges that must be overcome to develop a valid molecular biomarker of the heat-adapted state, and highlighting future research opportunities.

## Introduction

Humans are often required to operate and perform in hot and or humid environments. Compared to a cool environment, these conditions reduce physical work capacity [52], impair performance [69, 90] and increase the risk of heat-related illnesses [9]. Unfortunately, due to the warming effects of climate-change, exposure to this type of environment is likely to increase in the future [41]. However, repeated frequent exposure to environmental conditions that are sufficient to elicit pronounced thermo-physiological strain result in within-life phenotypic adaptations that improve an individual’s ability to maintain thermal homeostasis when subsequently exposed to a hot environment [38]. This process is termed heat *acclimatisation* when occurring in a natural environment and heat *acclimation* when undertaken in a controlled (laboratory) environment [84]. Heat acclimatisation/acclimation (HA) is an effective intervention for mitigating decrements in physical work capacity and performance in the heat [16], and reducing the risk of heat-related illnesses [183]. As such, HA is widely advocated for individuals operating in these conditions [160, 170].

The phenotypic adaptations to heat are commonly quantified by exposure to a standard exercise-heat stress, often termed a ‘heat acclimation state test’ (HAST) [169, 211], where the physiological outputs (e.g. sweating rate, cutaneous blood flow) and disturbances (e.g. skin and deep body temperature) to a fixed thermal input (e.g. ambient thermal stress and work rate) [164] are measured, with deep body temperature regarded as paramount due to its association with heat illness [9] (see Sect. “The heat-adapted phenotype” for more detailed discussion). However, quantification of these physiological indices requires carefully controlled environmental conditions (e.g. environmental chamber), work rate standardisation and specialist equipment, can be invasive (e.g. measurement of deep body (rectal or oesophageal) temperature), may lack objectivity, and is typically based upon change scores (i.e. pre vs. post comparisons) rather than absolute values. Less-invasive deep body temperature measurements (e.g. sub-lingual, axillary, tympanic) lack validity [136], whilst the utility of other non-invasive physiological indices of heat adaption (e.g. cardiovascular indices) has only been evidenced under carefully standardised environmental conditions [49, 202]. Together these factors can negate the practical utility of implementing a HAST, particularly in contexts where large numbers of personnel may need to be evaluated on a short time scale and with significant logistical constraints (e.g. defence [160]) and limit the use of physiological indices of heat adaption (e.g. assessment of thermoeffector outputs and physiological disturbances) in circumstances where the input conditions are not strictly controlled.

A biomarker is ‘*a defined characteristic that is measured as an indicator of normal biological processes, pathogenic processes or responses to an exposure or intervention*’ [45], p. 43). Biomarkers can be derived from histologic, radiographic, physiological, or molecular characteristics [21], with the latter having biophysical properties, which allow their measurements in biological samples such as plasma, urine or sweat [107]. Within the context of HA, a valid molecular biomarker may negate the need for a HAST, or reduce the technical requirements and reliance on the assessment of ‘hallmark” physiological indices of adaption, i.e. physiological biomarkers [164, 165], and enable an evaluation of whether an individual had acquired a heat-adapted phenotype through the sampling and assessment of a biological medium. Such a molecular biomarker may have important utility in circumstances where the assessment of physiological biomarkers is not viable, where reliable molecular markers of adaptation could not only provide assurance of the efficacy of HA, but also reduce heat illnesses and lead to more personalised approaches to adaptation [160]. Although, to date, such a molecular biomarker has not been identified, technological advances in non-invasive and minimally invasive biosensing [60, 158], low-cost easy-to-use analyte measurement approaches [88], continuous and remote monitoring wearable technology [184], and multi-molecule biomarker development [48], make identifying molecular biomarkers of heat adaptation a timely research challenge.

We commence this narrative review by briefly reviewing the thermo-physiological adaptations occurring with HA, including both the whole-body and cellular adaptations that are the ‘hallmark’ physiological characteristics of the heat-adapted phenotype. We subsequently review the literature that has examined changes in a number of candidate biomarkers within the context of HA, with a focus on molecules implicated in aspects of the adaptive response to heat, including fluid regulation, energy homeostasis, and sympatho-adrenal balance, as well as indices of inflammation, sudomotor adaption and acquired thermal tolerance. We conclude by highlighting a number of challenges that future research will need to overcome in order to develop a valid biomarker of HA, as well as future research opportunities that may prove to be fruitful in the development of a biomarker of HA.

## The heat-adapted phenotype

The adaptations occurring with HA have been extensively described previously and the reader is guided to reviews by Périard et al. [164, 165] for detailed descriptions. Briefly, repeated daily heat exposures of at least 60 min duration at an intensity sufficient to elevate deep body temperature and skin temperature to a level eliciting pronounced sweating invokes a multi-system array of adaptions occurring over different time courses [53]. Within 4 to 5 days a pronounced increase (> 5%) in plasma volume is observed (e.g. [14, 143, 185]), mainly underpinned by the fluid conserving effects of the hormones aldosterone and arginine-vasopressin (e.g. [2, 54, 140, 146]) as well as an oncotic effect from intravascular protein influx [162, 185]. The resultant hypervolemia increases stroke volume and, possibly in conjunction with a reduction in sympathetic nervous system excitability [81, 83, 192], results in a lowering of heart rate and improved cardiovascular stability [216].

An increased sweating rate can be detected from the second day of HA, but it is generally believed that > 10 days is required to obtain the complete changes in the sweating threshold and gain (sensitivity) and sweat electrolyte concentration that constitute ‘full’ sudomotor adaptation [175, 210]. The threshold for cutaneous vasodilatation is also reduced [175], which enables greater internal heat transfer as a result of increased convective heat transport from the ‘core’ to skin, whilst the sudomotor adaptations support increased wet-heat transfer from the skin to the environment (under conditions permitting evaporation). There is also some evidence for a shift towards a more metabolically efficient phenotype, although these data are primarily derived from long-term HA studies of rodents [131, 132]. Nevertheless, although it has been highlighted that the research is equivocal [29], a number of human studies employing typical HA durations (e.g. 7–14 days) have shown an improved metabolic efficiency during exercise following HA (e.g. [181, 188, 224]), with some suggesting that the resultant reduction in metabolic heat production plays a substantial role in the lower thermal strain that is evident following an HA intervention [177].

HA also induces a range of cellular adaptions that confer improved cytoprotection [139]. This acquired thermal tolerance (ATT) appears to share a common pathway with the systemic adaptations to HA that is mediated by heat shock proteins (HSP) [104], a conserved group of proteins that serve as molecular chaperones and accelerate cellular repair from heat stress, ischaemia and endotoxic shock [101, 108]. Compared to our understanding of the time course of the systemic adaptations to heat, understanding of the intracellular adaptations is relatively limited. Nevertheless, a recent meta-analysis suggests that the number of days of HA is a significant moderator of the intracellular HSP (iHSP) response [142]. This is exemplified by data from Marshall et al. [122] who showed that iHSP levels in peripheral blood mononuclear cells (PBMCs) were unchanged following two consecutive daily 2-h bouts of exercise-heat stress, and Yamada et al. [218] who reported elevated baseline iHSP levels from day 6 through to day 10 during a 10-day (100 min day^−1^) HA program.

Together, the multi-systemic adaptations occurring with HA, including expanded plasma volume, reduced heart rate, and enhanced skin blood flow and sweating, result in a lower skin and deep body temperature and an improved perceptual response, and in conjunction with ATT, constitute the ‘hallmark indices’ of the heat-adapted phenotype [164, 165].

## Candidate biomarkers

It is well established that acute exposure to a hot environment that is sufficient to elevate thermal strain also elicits a pronounced neuroendocrine response [125]. Likewise, the role of the neuroendocrine system in the long-term adaptation to heat has long been recognised [27, 148], as has the multi-systemic nature of adaption to heat and the interconnection between the systems controlling thermoregulation and those regulating fluid balance (e.g. renin–angiotensin–aldosterone system), energy homeostasis (e.g. hypothalamic–pituitary–thyroid axis [200]) and sympatho-adrenal activity (e.g. sympathetic–adrenomedullary and hypothalamic–pituitary–adrenal axis) [83]. Accordingly, the initial focus of this section is the evaluation of research that has measured the concentration of molecules that are associated with these physiological systems, or related processes, in response to HA. Given the notable thermoregulatory differences between species [225], we generally constrain our review to literature examining changes in the concentration of relevant molecules in the biological samples of humans undertaking HA, and the consideration of candidate biomarkers where there is a biologically plausible rationale for their measurement. We subsequently extend the scope of our review to encompass research that has examined changes in inflammatory biomarkers with HA, and the measurement of putative heat adaptation biomarkers in other biological mediums, including the measurement of intracellular heat shock proteins, as well as use of sweat biomarkers.

### Fluid balance and regulation

Adaption to heat causes an array of phenotypic alterations that improve the regulation of water and electrolyte balance, including an increase in total body water and plasma volume expansion, and a reduction in electrolyte losses [22]. These adaptations are primarily controlled by the action of the renin–angiotensin–aldosterone hormone system [8]. In response to a decreased perfusion of the juxtaglomerular apparatus in the kidneys, renin is released, which enables the conversion of angiotensinogen to angiotensin-I and subsequently to angiotensin-II by the action of angiotensin converting enzyme [8]. Angiotensin-II causes constriction of resistance vessels to elevate blood pressure, reabsorption of sodium in the proximal tubules of the kidney, and the release of aldosterone by the adrenal gland and arginine-vasopressin (AVP) from the posterior pituitary gland; the latter is also released in response to hyperosmolality detected in the pre-optic anterior hypothalamus as well as in response to baroreceptor firing from hypovolemia [79, 207]. Aldosterone acts on the mineralocorticoid receptors in the distal tubule and collecting duct of nephrons in the kidney, directly impacting sodium absorption and potassium excretion, resulting in elevated plasma sodium and the osmotic retention of water in the blood, whereas AVP mainly acts on aquaporin channels in the kidney tubules increasing water reabsorption [79]. Plasma protein levels may also play a role in the expansion of plasma volume through an oncotic effect that may be mediated by the combined actions of the lymphatic system [155], increased albumin synthesis [221] and reduced total albumin losses [76], although this appears to be a supportive rather than dominant mechanism [162]. The potential utility of a range of a number of putative biomarkers associated with these processes is discussed below.

#### Aldosterone

Although plasma aldosterone concentration increases may occur in response to intense exercise performed in temperate conditions [115], this effect is potentiated during exercise in the heat [135] and with hypohydration [56]. When plasma aldosterone is measured following a standard exercise-heat exposure (i.e. HAST) the aldosterone concentration is often shown to be diminished following an HA intervention (e.g. [54, 61, 96, 146, 189, 191]), likely as a result of increased sensitivity to the effects of aldosterone to changes in plasma osmolality [22]. However, this effect is no longer evident 1 week after HA [61] and the decline in plasma aldosterone during a post-HA HAST has not been observed in all studies (e.g. [10, 47, 100, 147]). Moreover, as detailed in Sect. “Introduction”, the practical utility of undertaking a HAST may be limited in many contexts [160], and baseline changes in the concentration of fluid regulatory hormones are likely to be more relevant in terms of a viable candidate biomarker of HA.

A number of studies have shown a significant increase in the resting baseline concentration of plasma aldosterone following an HA intervention [54, 143, 146, 147]. This is exemplified by Nielsen et al. [146] who demonstrated that mean (SD) baseline plasma aldosterone concentration increased from 96.6 (12.8) pg mL^−1^ to 156.0 (15.3) pg mL^−1^ following 9–12 consecutive days exercise in dry heat (90 min day^−1^), and Francesconi et al. [54] who demonstrated a significant increase in baseline plasma aldosterone concentration from ~ 12 to 17 ng dL^−1^ following a 10-day HA intervention (100 min day^−1^) in a combination of hot-dry and hot-wet environments. This effect of HA on baseline aldosterone does not appear to be significantly affected by the superimposition of modest dehydration (average daily body mass loss − 2.71 kg) during the HA regime [143], but may be affected by the level of dietary sodium, with high dietary sodium consumption associated with an abolished aldosterone response and low dietary sodium associated with a potentiated aldosterone response [2]. However, it is important to note that some studies have shown unchanged (e.g. [10, 47, 61, 96, 100, 189, 191]) or even reduced [161] baseline plasma aldosterone concentration with HA. It has been speculated that these apparently divergent findings may be related to differences in sodium balance, as well as different experimental models, including the mode and method of HA and training status of the participants [22], seasonal effects on aldosterone levels have also been reported [47, 94], whereas the extent to which systemic aldosterone concentration reflects the concentration at the renal mineralocorticoid receptors is unclear. Nevertheless, a recent meta-analysis concluded that, although there was high inter-investigation variability, there was a small significant increase (+ 25 ± 35%) in baseline plasma aldosterone concentrations following HA [205]. However, at present there is a paucity of data examining the time course of changes in resting aldosterone concentration with sufficient resolution over the induction and decay of HA and an increased understanding of the temporal relationship between plasma aldosterone and the plasma volume changes occurring with HA will be necessary to establish the utility of aldosterone as a HA biomarker.

#### Arginine vasopressin

It is generally accepted that, similar to plasma aldosterone concentration, plasma AVP concentration is acutely increased during intense exercise [79], but during exercise in the heat significant increases in plasma AVP are observed at lower work rates [64]. There is some evidence that the magnitude of increase in plasma AVP during exercise in the heat is diminished following HA [64, 146], although conversely, others studies have suggested that the increase in AVP might be larger following HA [147]. Similarly, Mudambo et al. [140] demonstrated that resting baseline plasma AVP levels were significantly elevated (+ 61%, *P* < 0.001) in 24 soldiers following 30 days of field-based HA, but interventions of this duration are uncommon and this finding appears to be in the minority, with the majority of studies suggest that baseline plasma AVP levels are unchanged following an HA intervention (e.g. [61, 64, 146, 147, 161]). Overall, these data are consistent with a recent meta-analysis, which concluded that there was no effect of HA (− 5 ± 15%) on resting plasma AVP concentration [205]. Nevertheless, this conclusion, as well as the apparently conflicting findings from different studies may, at least in part, be a consequence of the short half-life of AVP (< 30 min), whilst the fact that AVP is unstable, even in isolated plasma [190], combined with its small size, means it cannot be measured by sandwich immunoassay, but only by less sensitive competitive immunoassays [137].

#### Copeptin

Given the challenges posed by plasma AVP measurement there has been a growing interest in the potential of surrogate measures of AVP concentration [190]. Copeptin is a 39-amino acid glycopeptide that forms the c-terminal part of the AVP precursor protein preprovasopressin [137]. It is released in an equimolar fashion with AVP from magno- and parvo-cellular neurons of the hypothalamus and parallels the increase in AVP in response to alterations in tonicity and blood pressure [25]. However, in contrast to AVP, copeptin has a longer half-life [15], is stable in serum or plasma for days [137] and is less technically challenging to assay [25]. Together these factors may overcome some of the issues associated with the measurement of AVP.

Stacey et al. [190] examined the plasma copeptin concentrations in humans in response to thermal stress, demonstrating a significant rise in copeptin [10.0 (6.3) vs. 16.7 (9.6) pmol L^−1^, *P* < 0.001] among a group of 15 soldiers undertaking a 3.5 h simulated combat assault in a hot environment (27 °C), as well as evidence of a threshold effect, indicated by a negligible copeptin increase in individuals who did not exceed a deep body temperature of 38 °C. Moreover, the post-assault copeptin concentration was strongly related to the plasma osmolality (*r* = 0.70, *P* < 0.01), as well as the accumulated thermal stress (area under the curve for a deep body temperature > 37 °C; *r* = 0.78, *P* < 0.01). In a subsequent study, Stacey et al. [191] measured copeptin in a group of 23 soldiers over a 23-day natural HA intervention and demonstrated that neither the resting baseline, nor post-HAST, copeptin concentration changed over the HA period, although there was a rightward shift in the serum osmolality–copeptin relationship following HA. In a study by the same group, Omassoli et al. [156] again demonstrated that baseline copeptin was unchanged over the course of the 23-day HA intervention—a finding that has since been corroborated by another research group [152] employing a prolonged HA intervention (~ 28 × 60 min exercise-heat stress exposures over 5.5 weeks). However, in contrast to their previous study, Omassoli et al. [156] also reported that the post-HAST copeptin concentration was reduced over the course of the HA intervention, but their analyses do not clarify when the reduction in copeptin became significant, although the nadir value occurred on day 9 of the 23-day HA intervention.

At present, the reason for these discrepant findings are unclear, particularly given the commonalities between the studies. Moreover, plasma copeptin concentration appears to be affected by a number of non-thermal stimuli and disease conditions [137] and may therefore lack sufficient sensitivity and specificity to be utilised as a biomarker of HA. Thus, at present, the available research does not support the measurement of resting plasma copeptin concentration as a viable biomarker of HA, although there may be some utility if plasma copeptin is assessed following a standard HAST. Further research is needed to reconcile discrepancies between the limited number of available studies and to examine plasma copeptin levels over more common HA durations (e.g. 8–10 days; [205]) than those that have been studied to date (e.g. 23–30 days; [152, 156, 189, 191]). In addition, future studies measuring copeptin concentration should consider the fact that, while the longer half-life relative to AVP provides greater measurement stability [15], this could result in a temporal dissociation between these molecules in situations eliciting rapid changes in AVP concentration.

#### Renin

An increase in plasma renin concentration has frequently been demonstrated following exercise-heat stress [10, 33, 47, 54, 146, 147], with some evidence that the magnitude of increase in plasma renin may be attenuated following HA [47, 146] and that this effect may be greater than for other fluid regulatory hormones such as aldosterone [54], but this has not been shown to occur consistently [10, 33, 64, 146]. The reasons for these equivocal findings are not immediately clear, although Francesconi et al. [54] reported a more pronounced attenuation of the increase in plasma renin levels following HA when HASTs were performed in the hypohydrated state, compared to the euhydrated state, which was attributed to the combined effects of expanded plasma volume, attenuated renal vasoconstriction and a decreased sympathetic response to exercise-heat stress when heat adapted. Therefore, as has been described for other hormones involved in fluid regulation [140], variations in hydration levels may affect the influence of HA on renin levels during a HAST, with effects generally appearing to be more pronounced when hypohydration is superimposed on the exercise-heat stress.

Based upon the data of Finberg and Berlyne [47], Davies et al. [33], Armstrong et al. [10] and Nielsen et al. [146] a recent narrative review concluded that the baseline plasma renin concentration is unaffected by HA [22]. However, the data from Finberg and Berlyne [47], which demonstrated a small but statistically significant reduction in resting plasma renin concentration following a 7-day HA intervention, may have been mis-interpreted. Similarly, data from Nielsen et al. [147], which claimed to show an increase in resting renin concentration with an 8 to 13-day HA regime consisting of daily exercise to exhaustion in hot-humid conditions, were not included in this review, although within this study there appears to be discrepancy between the text conclusion and the data presented.

Therefore, the data are again somewhat equivocal for the effect of HA on resting and post-HA HAST plasma renin concentration. Whilst protocol differences may influence these findings, it is worth noting that in a number of the studies demonstrating a null effect (e.g. [33, 146]), data were obtained from a limited number (≤ 6) of participants, which will have resulted in low statistical power and an increased likelihood of Type II error.

#### Atrial natriuretic peptide and related hormones

Atrial natriuretic peptide (ANP) is a small peptide that is secreted by the atria of the heart as result of atrial distension due to a high systemic blood pressure. ANP counteracts some of the effects of aldosterone, decreasing reabsorption of sodium from the inner medullary collecting duct and increasing urinary sodium excretion, as well as increasing vascular protein efflux through effects on capillary permeability [214], resulting in a decreased plasma volume [31]. Pro-ANP is the precursor to ANP, whereas brain natriuretic peptide (BNP) is excreted by the ventricular cardiomyocytes and exerts similar effects to ANP, but has a longer half-life and lower receptor biding affinity [141, 197], with both binding to natriuretic peptide receptor A to induce cyclic guanylyl monophosphate as a second messenger in the target cells [214]. Despite their role in fluid regulation, to date, very few studies have examined changes in plasma ANP, pro-ANP, and BNP levels following HA in humans. Mudambo et al. [140] demonstrated a small, statistically significant, reduction in resting plasma ANP concentration following HA, whereas plasma BNP was unchanged although, as mentioned previously, this study employed an atypical duration of HA (30 days *c.f.* 8–10 days) and was undertaken in a field setting. In contrast, Kraemer et al. [100] reported no changes in baseline plasma ANP over an 8-day HA intervention, and Patterson [161] reported no statistically significant difference in resting baseline plasma ANP among 12 individuals after either 8 days or 22 days of an HA intervention, with a numerical *increase* of 32% apparent on day 22, which might be reflective of the sustained hypervolemia induced by their experimental model [162]. Again, these divergent findings likely stem from differences in the types of HA intervention in these studies, which include natural and laboratory HA, different thermal forcing functions (e.g. controlled hyperthermia, fixed work rate) and various durations, amounts of exercise, and dietary and hydration conditions. Pro-ANP does not appear to be affected by HA [152], but has received limited attention in the context of HA.

#### Other fluid regulatory molecules

Other molecules that are involved in the control of fluid balance and may play a role in the adaptive responses seen with exposure to heat have received relatively little attention; we are unaware of any research that has examined the effects of HA on the resting plasma concentrations of angiotensinogen, angiotensin-I and angiotensin-II in humans. Changes in the plasma concentrations of these molecules have been documented during acute exposure to heat [99], but cross-sectional observational research suggests that there does not appear to be a seasonal effect on baseline angiotensin-II, even when these is some evidence of acclimatisation [94]. Nevertheless, given the involvement of these molecules in the fluid-regulatory pathways that are linked to aspects of the heat-adapted phenotype, future investigation of these molecules may prove fruitful in the context of a biomarker of the heat-adapted state, although their utility could be limited by extremely short circulating half-lives [3, 80] and susceptibility to changes in hydration state [168].

#### Markers of kidney function and kidney injury

Heat stress represents a profound challenge to kidney function and can increase the risk of acute kidney injury (AKI) [182], which is augmented with increasing thermal strain and dehydration [23]. A number of recent studies have examined the effects of HA interventions on biomarkers of kidney function and AKI to establish if there is protective adaptation of the kidney with repeated heat exposure; biomarkers evidencing such an effect may have utility in identifying the heat-adapted phenotype. Based upon increases in serum creatinine and reductions in estimated glomerular filtration rate (calculated from serum creatinine concentration), Pryor et al. [167] reported that 75% of individuals undertaking 2 h of exercise in the heat exceeded the clinical threshold for AKI before a 4-day HA intervention, with only 58% of individuals meeting this threshold after the HA intervention. However, neither the mean baseline nor the mean post-exercise serum creatinine concentration were significantly altered by the HA intervention. Similarly, Omassoli et al. [156] reported a reduction in the number of individuals attaining serum creatinine levels exceeding the threshold for clinical AKI from 14/20 to 1/20 individuals following 23 days of HA. However, in contrast to Pryor et al. [167], a reduction in mean serum creatinine was also reported following a standard HAST, although baseline serum creatinine was unchanged. In a subsequent study, Ravanelli et al. [171] reported that neither glomerular filtration rate (calculated as the product of urine creatinine concentration and urine flow rate divided by serum creatinine concentration) nor its constituent analytes were altered by a 7-day passive HA intervention.

More recent research examining the effects of HA on AKI [73] has assessed the urine concentrations of neutrophil gelatinase-associated lipocalin (NGAL), a biomarker of renal tubular damage [133] and urinary kidney injury molecule-1 (KIM-1), a transmembrane glycoprotein that indicates proximal tubule injury [72]. This research indicated that urinary NGAL was unaffected by a HAST and did not differ pre- to post-HA, whereas KIM-1 was elevated following the HAST, but to a similar extent before and after HA, with no change in baseline concentration [73]. Thus, whilst there is some limited evidence that HA may reduce the achievement of serum creatinine levels associated with AKI during a HAST, and that longer HA interventions may lower the post-HAST serum creatinine concentration, other biomarkers of AKI (e.g. NGAL, KIM-1) appear unaffected by HA. Moreover, none of these biomarkers of kidney of AKI have shown baseline changes following HA, indicating that they may have limited utility in the assessment of the heat-adapted phenotype. Finally, it is important to note that, to date, no studies examining the effect of HA on AKI have assessed urine concentrations of insulin-like growth factor binding protein 7 (IGFBP7) and tissue inhibitor of metalloproteinase 2 (TIMP-2), which appear to outperform other biomarkers of AKI in clinical settings [95]; these biomarkers should be considered in future research examining renal adaptions to HA.

#### Haematological indices associated with altered fluid balance

The hypervolemia that is a characteristic of the fluid shifts occurring with the heat-adapted phenotype typically results in a pronounced haemodilution, leading to a reduction in both the blood haematocrit (volume percentage of red blood cells in blood) and blood haemoglobin concentration [17, 74]. Because this hypervolemia is partly driven by an oncotic effect, to which albumin is the biggest contributor [180, 185], the expanded intravascular volume is also often associated with an increase in the plasma protein and plasma albumin content [162, 185]. This effect may become evident after 2 days of HA [185] and has been shown to persist for as long as 22 days when a controlled hyperthermia HA regime is used [162]. Although some studies have not reported an increase in plasma protein content with HA (e.g. [17]), this may be due to differences in the method of expression, with changes in total protein content typically more pronounced than changes in protein concentration [162], presumably because the latter acts as the stimulus for the plasma volume expansion. As a consequence, these simple haematological parameters may have some utility in identification of the heat-adapted state, but their time course of acquisition may precede the development of other aspects of this phenotype such as sudomotor changes [164]. Moreover, whilst within-individual reductions in haematocrit and haemoglobin concentration are consistently observed following an HA intervention [17], there is pronounced inter-individual variability in the baseline haematocrit and haemoglobin, which may be related to a range of physiological (e.g. sex [20]; training status [145], genetics [119]), environmental (e.g. hypoxic exposure[114]) and nutritional factors (e.g. hydration [26], protein and carbohydrate consumption [154]).

Similarly, recent research has shown that a prolonged HA regimen (5.5 weeks) can result in an increased haemoglobin mass [152], although this does not appear to be the case with shorter HA regimens [172], and the effects of HA on erythropoietin levels are equivocal [152, 172]. Moreover, plasma albumin and protein levels can increase with training intensification, independent of HA [152], and may be altered by dietary protein consumption [67]. Each of these factors could adversely impact on the utility of these simple haematological indices as a biomarker of HA and suggests that, in isolation, these measures may not be appropriate, but they may provide valuable information in situations where it is possible to control for the aforementioned potential confounders.

### Energy homeostasis

Although the role of energy homeostasis in thermoregulation is well accepted [200], it has mainly been studied within the context of exposure to cold, and in the control of the production of additional heat via the influence of hypothalamic-pituitary-thyroid axis on the metabolic processes that constitute facultative (non-shivering) thermogenesis [195]. Similarly, the role of the prostaglandin–cyclooxygenase system is well established in febrile temperature regulation [86] and has recently received some attention in the context of exposure to cold [43, 50]. However, there is some evidence suggesting that these processes may also play a role in the adaptive processes to chronic heat exposure and as such may be suitable for further investigation in the context identifying a biomarker of the head adapted phenotype.

#### Thyroid hormones

Thyroid hormones play a central role in the control of heat generation from biological processes that are inherent to homeothermic species, including humans [87, 195]. The circulating levels of the thyroid hormones triiodothyronine (T3) and thyroxine (T4) are regulated by the hypothalamic–pituitary–thyroid axis. In response to low T3 and T4 levels the hypothalamus releases thyrotropin-releasing hormone (TRH), which stimulates the release of thyroid stimulating hormone (TSH) from the anterior pituitary. This results in the secretion of thyroid hormones from the thyroid gland (primarily T4) and the further production of the more potent T3 through the action of deiodinase on T4 in peripheral organs, including the liver. T3 and T4 are both active in their ‘free’ state (i.e. unbound to thyroxine-binding globulin, transthyretin, or albumin), and inhibit TRH and TSH, thus creating a negative feedback loop which regulates circulating levels [187]. T3 and T4 have multifarious effects including on development [130] and cardiovascular function [44], as well as on body temperature regulation [194], the latter appears to be mainly related to effects on metabolic rate and non-shivering thermogenesis [87, 195]. Nevertheless, more than 50 years ago Weiner and Collins [27] suggested that ‘*a reduction in thyroid activity in hot conditions might play a part in maintaining thermal balance by bringing about a slowing in metabolism and heat output*.*’* (p. 790). Subsequent animal research demonstrated that rats treated with propylthiouracil to lower circulating T3 and T4 have a lower deep body temperature at rest and during heat stress [219], and that after a 4-week heat acclimation intervention blood T3 and T4 levels were spontaneously reduced in rodents [132]. These changes in thyroid hormone levels appeared to be causally related to the development of a more metabolically efficient phenotype during HA, as determined through measurements of cardiac mechanics, with the adaptations blunted when propylthiouracil was administered to maintain a euthyroid state throughout the HA [131], although it remains to be established if the improved metabolic efficiency reported after some human HA studies (e.g. [181, 188, 224]) is related to the action of thyroid hormones.

In humans, cross-sectional analysis of a large data-set of deep-body temperature measurements demonstrated a linear relationship between TSH levels and resting deep-body temperature [153]. A recent meta-analysis of studies in healthy euthyroid adults evidenced significant seasonal variation in thyroid hormones, including lower circulating TSH and T3 levels during the summer compared to the winter [105]. Beyond these cross-sectional observational studies, a limited number of studies have measured thyroid hormone levels in humans over the course of a HA regimen. An early study conducted by Sridharan et al. [196] reported a reduction in resting plasma T3 and T4 after 4, 2-h daily heat exposures (45 °C, 30% R.H.), accompanied by a reduction in tympanic temperature and increased sweating rate, with further reductions in plasma T3 evident after the 8 day of heat exposure. More recently, in an unpublished study, McIntyre [128] demonstrated that the change in resting plasma total T4 after a 6-day HA was related to the magnitude of the accumulated endogenous thermal ‘dose’ (total area under the curve for rectal temperature > 38.5 °C,* r* =  − 0.32, *P* = 0.021), with a larger endogenous thermal dose resulting in a greater reduction in plasma total T4. In a subsequent study examining two different 12-day HA regimens, the same group demonstrated a significant reduction (*P* = 0.006) in resting plasma free T3 following a 12-day hot water immersion HA regimen [129], but no significant change in plasma T4 levels. Interestingly, in unpublished data from this group [128], it appears that the plasma free T3 level was related to the reduction in resting rectal temperature (*r* = 0.47, *P* = 0.044), as well as the endogenous thermal dose accumulated during a 12-day HA regimen (*r* = − 0.57, *P* = 0.017).

Whilst these data suggest that the measurement of thyroid hormones may have utility in the assessment of heat-adapted phenotype, some caution is warranted. For instance, McIntyre [128] noted that the reduction in rectal temperature preceded the change in thyroid hormone levels during HA, indicating that either the thyroid hormones were not causally linked to the reduction in deep body temperature, or that they are only mechanistically relevant for the longer-term adaptive response. Moreover, circulating plasma T3 may be reduced after a single heat exposure [42], raising the possibility that measurements made in close proximity to an acute heat exposure could be similar to those found in the resting state following chronic heat exposure, but without incurring the adaptive benefits. There is also some evidence of sex differences in the thyroid hormone responses to a thermal stimulus [105], whilst non-thermal factors including calorific restriction [37], stress [78] and disease [89] also affect thyroid hormone levels and may therefore impact on their utility as a biomarker of heat adaptation.

#### The prostaglandin–cyclooxygenase system

Prostaglandins are potent bioactive lipid messengers belonging to the eicosanoids, a family of oxygenated 20 carbon fatty acids [111, 159]. There are four principal bioactive prostaglandins generated in vivo: prostacyclin (PGI_2_), prostaglandin D_2_ (PGD_2_) prostaglandin E_2_ (PGE_2_) and prostaglandin F2α (PGF2α) [174] with PGE_2_ the most common [111]. Prostaglandins are synthesised from arachidonic acid in most mammalian tissues via the actions of prostaglandin synthases and two cyclooxygenase (COX) isoenzymes: COX-1, which preferentially oxygenates exogenous arachidonic acid and is responsible for regulating baseline prostaglandin levels and mediates a range of ‘housekeeping’ functions, such as gastric epithelial cytoprotection and homeostasis [174]; and COX-2 which acts on endogenously produced arachidonic acid and controls prostaglandin synthesis in response to inflammation [159].

PGE_2_ is a principal pyrogenic mediator of the elevation in core temperature occurring with fever [86]. The upregulation of PGE_2_ during fever is functionally coupled to the inducible COX-2 isoform [178] with the first febrile-phase initiated by PGE_2_ originating in peripheral tissues and the subsequent phase caused by the binding of PGE_2_ to a specific EP3 receptor in the median pre-optic area to affect hypothalamic neurons that regulate thermoregulation. Although the role of this pathway in non-febrile temperature regulation is less well established, COX-2 gene deficient mice are unable to effectively defend their deep body temperature during cold exposure [116]. Research in humans has shown a thermogenic response of the COX-2/PGE_2_ pathways during acute (120 min) cold exposure in humans and a relationship between rectal temperature and PGE_2_ during acute exposure to cold and thermoneutral conditions, although no effects were evident during acute exposure to hot (40 °C; 40% R.H.) conditions [43]. Similarly, administration of acetaminophen, a COX enzyme inhibitor, reduced core temperature of humans during an acute (120 min) passive exposure to 20 °C, 40% R.H [51] as well as during running [19] and cycling [126] exercise in the heat (30 °C, 50% R.H.); this latter finding has been challenged [28], whilst studies that have used forearm dermal microdialysis of COX inhibitors indicate that both COX isoforms may be involved in sudomotor regulation in the heat [58], although in contrast to studies of oral COX inhibitors (which may influence central and peripheral thermoregulatory mechanisms), microdialysis administration will only exert peripheral effects.

Given the purported role of COX/PGE_2_ pathway on temperature regulation it is surprising that, as far as we are aware, only one study has examined these molecules in humans undertaking HA. Shin et al. [198] demonstrated that both serum PGE_2_ and COX-2 were significantly reduced (− 18.5% and − 17.3%, respectively) following a 3-week HA intervention, with both of these molecules explaining a significant proportion of the variance in mean body temperature (PGE_2_ *r*^2^ = 0.495; COX-2 *r*^2^ = 0.369). Interestingly, the concentration of serum orexin, a neuropeptide that is produced in the hypothalamus [34], was also reduced (− 17.7%) and was also related to the variance in mean body temperature (*r*^2^ = 0.382). Orexin serves a number of functions, including regulating sleep and wake states, feeding behaviour, reward systems, mood and cognition [24], with evidence from animal studies indicating that orexin is also implicated in the regulation of body temperature at rest [134, 223] and during exercise [123]. However, whilst the findings of Shin et al. [198] are promising, it has been noted that this study did not adjust for the plasma volume changes occurring with HA and should therefore be interpreted cautiously [43]. Moreover, they are based on a limited sample size (*n* = 9), and an atypical HA regime consisting of 10 bouts of 30-min hot water immersion over a 3-week period, as well as deep and mean body temperature calculation from tympanic measurement which is of questionable validity (e.g. [1]). Therefore, although this HA regime was apparently effective at inducing hallmark induced of heat adaption (reduced deep body temperature and increasing sweating), it remains to be seen if these molecules demonstrate the same responses over more traditional HA regimens in air, when deep body temperature is assessed using gold standard method (e.g. rectal or oesophageal), and when plasma volume changes are accounted for.

### Sympathetic–adrenomedullary and hypothalamic–pituitary–adrenal axis

Evidence from HA studies suggests that the activity of the sympathetic–adrenomedullary (SAM) axis [46, 81, 118, 146, 156] and hypothalamic–pituitary–adrenal (HPA) axis [156, 192, 206, 208] may be altered in the heat-adapted state. The SAM and HPA axis work in coordination to regulate the ‘stress response’ over acute and chronic time scales [62, 204]. The processing of physiological stress signals by the brainstem and hypothalamus activates the neurocircuitry generating the SAM axis response, which is regarded as the most rapidly responding system of the stress response [62]. SAM activity is mainly mediated by the secretion of the catecholamines adrenaline and noradrenaline from the adrenal medulla and the release of noradrenaline from the sympathetic nerves [62], which initiates a range of physiological responses to prepare the body to respond to the stressor [204]. In contrast to the SAM axis, the HPA axis is a hormone system, which results in activation over a slower timescale than the SAM axis, but with more protracted effects [179]. Activation of the HPA axis is elicited by the paraventricular nucleus of the hypothalamus, which releases corticotropin-releasing hormone, and the arcuate nucleus which secretes growth hormone releasing hormone. Together this stimulates the secretion of adrenocorticotropic hormone and growth hormone from the anterior pituitary gland [7, 204]; AVP can also act synergistically with corticotropin-releasing hormone to stimulate adrenocorticotropic hormone secretion [106]. Thereafter, growth hormone binds to the growth hormone receptor and activates multiple downstream intracellular signalling cascades [7], whereas adrenocorticotropic hormone binds to receptors in the adrenal cortex of the adrenal gland, resulting in the release of the glucocorticoid hormone cortisol, which is the end hormonal product of the HPA axis [62, 204] and elicits a range of metabolic and immune functions that support the bodies response to stress [204].

#### Catecholamines

Sympathetic nervous system activity is implicated in thermoregulation during heat stress [150] and a change in SAM axis activity, typified by a reduction in sympathetic activity [81] and possibly increased parasympathetic activity [49], is characteristic of the heat-adapted state. Catecholamines have commonly been employed as molecular biomarkers of SAM axis activity [63, 68] and it appears that, compared to exercise in cool conditions, exercise in the heat results in an acute elevation in plasma catecholamine levels [46], with some evidence that this increase may become attenuated following HA [46, 81, 146, 166]. For example, Nielsen et al. [146] reported a significant increase in plasma adrenaline and noradrenaline concentrations during HASTs undertaken before and after 9–12 days of 90-min daily exercise-heat stress, with the concentration of both catecholamines reduced after 30 min of exercise in the post-HA HAST. Subsequent research has examined the time course of the reduction in plasma noradrenaline concentration following daily 90-min bouts of walking at 40% of maximal oxygen uptake in the heat, with the post-exercise reduction in noradrenaline becoming evident from the third day onwards and paralleling the time course of reductions in exercise heart rate [81]. Similarly, Febbraio et al. [46] reported that the plasma adrenaline concentration was reduced at the end of a 40-min HAST following a 7-day HA intervention, although noradrenaline levels during the HAST were not changed, whereas Nielsen et al. [147] reported no change in either plasma catecholamine following HA, although this study utilised an exercise to exhaustion HAST which lasted longer post-HA and may have masked any effects on catecholamine concentration. However, none of the aforementioned studies reported changes in the resting baseline concentrations of either plasma catecholamine.

Given the short circulating half-life of 1–2 min, as well as the influence of postural changes and the stress of venepuncture on plasma catecholamines [163], urine catecholamine measurements may be preferable in some situations, but they are influenced by the duration over which samples are obtained [68, 163] and do not offer the same temporal resolution as plasma measurements. Studies measuring urine catecholamine concentrations over the course of HA have reported conflicting results. Using an 8-day HA intervention consisting of 90 min day^−1^ of exercise-heat stress, Maher et al. [118] demonstrated that urinary noradrenaline excretion, assessed from samples collected from the onset of exercise to 1 h after exercise completion, was reduced during the last 3 days of the HA intervention, compared to the first 3 days. In contrast, Leppäluoto et al. [112] demonstrated no effect on resting urine adrenaline concentration (collected from 20:00 to 0800), but a slight increase in resting urine noradrenaline in participants exposed to dry heat (in a Finnish sauna; 80 °C) for 1 h, twice a day, for 7 days. The reasons for these conflicting findings are unclear, but should be interpreted cautiously given that potential analytical challenges have been identified in the assessment of urine catecholamines [68], the differences in temporal resolution for the urine samples between the studies, as well as notable protocol differences (e.g. active vs passive HA). In addition, Leppäluoto et al. [112] noted high individual variation in their data, and changes to fluid consumption practice mid-way through their study may have confounded their data.

#### Nephrines

Given the reported challenges with plasma and urine catecholamine measurement it has been suggested that metanephrine and normetanephrine, the o-methylated extra neuronal metabolites of adrenaline and noradrenaline, respectively, may offer appropriate alternatives for the measurement of sympatho-adrenal activity [39], overcoming many of the limitations associated with catecholamine measurement [163]. Plasma nephrine measures are more stable than those of the catecholamines [36] and demonstrate good correlation with both the plasma concentration and urinary excretion of their parent compounds [176]. However, to date, only a small number of research studies, from the same research group, have examined the concentration of plasma nephrines in response to HA. In a published abstract, Stacey et al. [189] reported that the post-HAST concentrations of plasma normetanephrine and metanephrine were both significantly reduced (*P* < 0.0001) following a 23-day natural HA (plasma normetanephrine: 948 (328) vs 461 (132) pmol L^−1^; plasma metanephrine: 302 (103) vs 230 (91) pmol L^−1^). Moreover, both the Δ normetanephrine and Δ metanephrine were correlated with the reduction in physiological strain index (calculated from deep body temperature and heart rate) over the HA period, which was interpreted as evidencing reduced sympathetic activation and led the authors to conclude that plasma nephrines could have utility as a biomarker of heat tolerance. In a subsequent study, Stacey et al. [192] demonstrated that the plasma free normetanephrine concentration was increased during a HAST, with the increase attenuated on day 6 and 23 of the intervention (but not on day 9) which, in conjunction with heart rate variability analyses, was interpreted as indicating a transition towards diminished sympathetic activity. In contrast, plasma free metanephrine did not increase over each HAST, although a significant main effect of HAST day was reported, but the location of this effect was not identified. Most recently, Omassoli et al. [156] reported significant main effects for both baseline and post-HAST free plasma metanephrine concentration over a 23-day HA, again, the locations of any effects were not reported, but the numerical nadir for the resting baseline free plasma metanephrine concentration occurred on day 2, whereas the post-HAST value declined progressively, reaching a nadir on day 23. However, it is important to note that there is high similarly between the methods of Stacey et al. [189], Stacey et al. [192] and Omassoli et al. [156], and these studies may be reporting data from the same participant pool, with possible overlap between the studies.

#### Cortisol

In addition to the measurement of plasma nephrines, Stacey et al. [189], Stacey et al. [192] and Omassoli et al. [156] each reported reductions in the post-HAST plasma cortisol levels over the course of their HA regimen, with Stacey et al. [192] indicating that this effect was significant from day 6 onwards and Stacey et al. [189] reporting a relationship between the Δ cortisol (*r* = 0.62) and the reduction in physiological strain index over the HA regime. These findings are consistent with a number of others studies that have also reported a reduction in post-HAST plasma cortisol levels following both passive (e.g. [112, 166]), and active (e.g. [10, 33, 201, 206, 208]), HA protocols. For example, Pilch et al. [166] reported that the rise in cortisol over a sauna was attenuated in the final exposure in a group of females undertaking 7 × 30-min saunas over a 14-day period. Similarly, Armstrong et al. [10] reported a significant increase in plasma cortisol levels during a HAST undertaken on day 1 of an 8-day active HA regimen, which was not evident on the final day. However, it is important to note that, with the exception of Tamm et al. [201], these studies reported no change in the resting baseline plasma cortisol levels, and whilst the majority of studies report reductions in post-HAST plasma cortisol following HA, some studies have shown no effect (e.g. [212, 213]). Although the reasons for these discrepant findings are not immediately clear, cortisol displays strong circadian effects [215] which may influence pre–post HAST measures. Additionally, hydration appears to potentiate the cortisol response to exercise-heat stress [30] and may also mediate the adaptive responses with HA; Francesoni et al. [55] reported that an increase in plasma cortisol during a HAST was only evident when participants were hypohydrated, but not when euhydrated, and that when the HAST was repeated in the hypohydrated state following a period of HA the increase in plasma cortisol was attenuated.

#### Other HPA-axis hormones

Compared to studies examining the cortisol response, a limited number of studies have measured other HPA-axis hormones in response to HA, with most focusing on adrenocorticotropic hormone (e.g. [112, 166, 201, 206]) or growth hormone (e.g. [55, 146, 147, 157]); we were unable to identify studies reporting changes in corticotropin-releasing hormone during HA in humans. Leppäluoto et al. [112] reported that plasma adrenocorticotropic hormone levels, measured post-sauna were unchanged over a twice-daily, 7-day sauna HA intervention, with similar data presented by Pilch et al. [166] when the same number of saunas were undertaken over 14 days. In contrast, Timpmann et al. [206] reported that plasma adrenocorticotropic hormone levels were attenuated during a HAST performed after an 8-day active HA, with similar data also reported by Tamm et al. [201] following a 10-day active HA. In each instance, baseline plasma adrenocorticotropic hormone levels were unchanged by HA. The discrepancies between these studies in the post-HAST changes may be related to differences in both the HA regimes (passive vs active) and HAST, with the exercise-heat stress approach employed by Timpmann et al. [206] and Tamm et al. [201] likely eliciting a significantly greater thermo-physiological strain than the passive sauna approach of Leppäluoto et al. [112] and Pilch et al. [166]. Circulating growth hormone levels are known to increase with hyperthermia [57] and thus a decrease in growth hormone concentration might be hypothesised following a period of HA concomitant with the reduction in deep body temperature. However, data on the effect of HA on growth hormone levels are similarly equivocal, with studies showing no effect following a HAST [146, 147], an attenuated response [157] or inconsistent findings [55], although these differences are not easily attributable to protocol differences given that all employed active HA and HAST approaches, they might be influenced by the pulsatile nature of growth hormone release [75]. Nevertheless, baseline plasma growth hormone responses were consistent, being unaffected by HA in each of these studies.

Taken together, the majority of studies suggest that HA does not have any substantial effect on resting baseline SAM or HPA-axis hormones, but may reduce the post-HAST levels, particularly with HAST approaches that elicit a pronounced thermo-physiological strain (e.g. active approaches). Given the established effect of an elevation in deep body temperature on SAM and HPA-axis hormone release [125], the post-HAST reductions in SAM and HPA-axis hormones following HA are likely reflective of attenuated thermo-physiological strain in the heat-adapted state. Interestingly, this effect does not appear to be evident in the resting state, despite the fact that resting deep body temperature is typically reduced with HA [164, 165], which may indicate a potential floor effect for these hormones. Therefore, SAM and HPA-axis hormones appear to have limited utility as a biomarker of the heat-adapted phenotype when only a resting baseline measurement is possible, but may have some utility in contexts where individuals can be exposed to a standardised thermal stressor, i.e. a HAST.

### Inflammatory markers

The most commonly measured inflammatory markers in the context of HA are the interleukins (IL), IL-6 and IL-10 as well as tumour necrosis factor (TNF) α, and C-reactive protein; other inflammatory markers have received scant attention. Interleukins are a type of cytokine that are expressed by leukocytes, as well as other cells, and regulate immune and inflammatory actions through multifarious actions on immune cells; including activation, differentiation, proliferation, maturation, migration, and adhesion. IL-6 is regarded as having both anti- and pro-inflammatory effects with IL-10 being considered an anti-inflammatory cytokine [91]. TNF α is an inflammatory cytokine produced by macrophages/monocytes during acute inflammation [85] and C-reactive protein is an acute phase reactive protein that is induced by IL-6 and has both pro- and anti-inflammatory properties [144].

#### Interleukin-6 and interleukin-10

The plasma concentration of both IL-6 and IL-10 has been shown to increase in response to an acute exercise-heat stress (e.g. [30, 70, 71, 104, 110, 173], although some studies have failed to report an increase [92, 149]. These differences between-studies could be the result of a number of factors including the magnitude of thermal stress that the participants were exposed to during the HAST [173], which is likely to be influenced by the HAST protocol as well as the health and fitness of the participants. IL-6 appears to be the most commonly investigated cytokine in the context of HA and whilst a number of studies investigating the effect of HA interventions on IL-6 have shown that neither the baseline nor the post-HAST IL-6 concentrations are different following a period of HA ([30, 71, 93, 213], others have indicated that plasma IL-6 concentration may be altered with HA. For instance, Lee and Thake [110] reported that the increase in IL-6 during exercise-heat stress was reduced on the 10th day of HA. Omassoli et al. [156] also reported significant main effects for both the baseline and post-HAST IL-6 concentration measured on days 0, 2, 6, 9 and 23 of HA, but the exact days when the differences occurred was not reported, which limits the conclusions that can be drawn. Similarly, although Kuennen et al. [104] reported that the immediate and 2 h post-HAST IL-6 data were unchanged following HA, there was evidence for a more-rapid return to baseline 2 h post-HAST. We were unable to identify any studies that have reported a reduction in baseline plasma IL-6 concentrations following a period of HA.

Compared to IL-6, a smaller number of studies have investigated the effect of HA on IL-10. Yamada et al. [218] showed no increase in IL-10 in HASTs undertaken before or after HA, whilst conversely, Kuennen et al. [104] demonstrated that the increase in IL-10 that was observed in an initial HAST was not evident after 7 days of HA, with similar data reported by Lee and Thake [110] on the 10th day of HA. Closer inspection of Kuennen et al [104] data suggests that following HA the baseline IL-10 concentration was approximately halved (from ~ 10 ± 1 to ~ 5 ± 1 pg^.^mL^−1^), but their statistical analysis, which does not appear to include a between group component, did not evaluate this. As far as we are aware, there are no studies that have reported a statistically significant reduction in baseline plasma IL-10 concentration. The apparent discrepancies between HA studies that have measured plasma cytokine concentrations can be explained by the research of Rhind et al. [173], which demonstrated that cytokine concentration increases during exercise are only evident when an individual becomes hyperthermic, and may be abolished when deep body temperature is clamped at resting levels during the same exercise task. Although the threshold deep body temperature for promoting the release of the various cytokines is unknown, these data provide a plausible mechanism accounting for the lack of increase in cytokine levels during the initial HAST in some studies (i.e. the thermal threshold for cytokine release was not surpassed) as well as the reduction seen in the post-HAST cytokine levels following a period of HA, where an elevation in cytokine levels was seen in the initial HA (i.e. the lower thermal strain during the HAST when heat adapted means that the individual no longer surpasses the thermal threshold for cytokine release).

#### Tumour necrosis factor α and C-reactive protein

Although TNF α levels have been shown to increase when exercise in undertaken in the heat compared to cooler conditions [193], a limited number of studies have reported plasma TNF α levels in response to HA. Both Kuennen et al. [104] and Lee and Thake [110] observed no increase in plasma TNF α concentrations in HASTs undertaken before or after HA, in each instance the HASTs employed were sufficient to elevate the concentration of other cytokines (e.g. IL-6, IL-10) suggesting that TNF α is less sensitive to heat stress than other cytokines. Similarly, Costello et al. [30] reported that C-reactive protein was unchanged in HASTs undertaken before or after 10 days of HA. Kaldur et al. [92] reported an in increase in the log C-reactive protein concentration during an endurance exercise task following HA, but the open-ended nature of their endurance task resulted in an increased test duration following the HA and limits the ability to compare these findings to other studies. In each of the aforementioned studies there was no evidence for baseline changes in the TNF α or C-reactive protein levels. This observation, as well as the high reported daily variability of a number of these inflammatory biomarkers [70], and the fact that inflammation is a generalised immune response which may be elicited numerous non-thermal stimuli, will likely negate the use of inflammatory markers in the context of a biomarker of the heat-adapted phenotype.

### Heat shock proteins

HSPs are a conserved group of proteins that vary in molecular mass from ∼ 15 to 110 kDa [101]. The expression of these proteins can be induced by a variety of stressors, including exercise, hypoxia, energy depletion, acidosis, reactive oxygen and high body temperatures [101, 103], with the 72-kDa protein (HSP72 or HSP70) appearing to be the most inducible and thermosensitive HSP [101], as well as the most studied HSP in the context of HA. Within cells these HSPs (iHSP) prevent apoptosis and inflammation [222] and act as a molecular chaperone, accelerating cellular repair from heat stress, ischaemia and endotoxic shock and providing cytoprotection against subsequent, more extreme, and potentially lethal exposure to these stressors [6, 101, 108]. HSPs can also be released into the systemic circulation from a variety of cells (eHSP), where they appear to serve a range of immunological functions [103].

#### Extracellular heat shock proteins

A number of studies have examined the effect of HA on extracellular HSP (eHSP) levels in plasma and serum, with the possibility of using eHSP as a biomarker of the HA phenotype proposed by Kresfelder et al. [102], who demonstrated that individuals who were able to effectively adapt to a 6-day HA regimen had a concomitant reduction in resting eHSP70. However, subsequent studies demonstrated mixed results. For instance, Yamada et al. [218] and Neal et al. [143] both reported that the resting baseline eHSP70 concentration was unchanged following 10–11 days of HA, with Yamada et al. [218] also demonstrating that eHSP levels did not increase in HASTs performed before and after the HA. In contrast, Marshall et al. [121] demonstrated a small post-exercise increase in eHSP on each of 2 consecutive days of exercise-heat stress, as well as a significant reduction in baseline eHSP70 levels on the day after the second heat exposure, but this likely proceeded the development of many of the whole-body indices of adaptation and so the physiological relevance of this change as a biomarker of HA is questionable. However, some studies have demonstrated that HA attenuates the eHSP response to a HAST. For example, Magalhães et al. [117] reported that although baseline eHSP70 was unaffected by an 11-day HA regime, a significant increase in eHSP70 observed in the HAST undertaken before HA was abolished in the post-HA HAST; a similar attenuation of the eHSP response to exercise-heat stress was reported by Lee and Thake [110] following a 10-day HA regime.

The reasons for the differences in eHSP response between these studies is not clear, although the data of Magalhães et al. [117] demonstrated that the increases in eHSP70 following a HAST had returned to baseline within 1 h, indicating that sample timing may influence findings. In addition, the eHSP concentration during exercise appears to be closely related to the peak deep body temperature achieved [117], which may need to exceed 38.5 °C to elicit an increase in eHSP70 levels [65]. Thus, taken together, it appears that there is some evidence for a post-HA reduction in eHSP levels when measured after a HAST, which may be a function of a lower deep body temperature when heat adapted, but this has not been universally demonstrated. However, baseline eHSP levels do not appear to be consistently reduced in the heat-adapted state. This conclusion is in keeping with the trivial effect-size of HA on resting eHSP70 (Hedges’ *g* = 0.18) reported in a meta-analysis [205]. Moreover, eHSP may also be elevated in inflammatory clinical conditions, unrelated to heat adaption (e.g. [109]), whilst the immunological functions of eHSP have led to the suggestion that it might have utility as a biomarker of overtraining [103] and data from Guy et al. [70] have shown a high within-participant variability in eHSP (37%) when measured within a 7-day period. Taken collectively, these factors may limit the sensitivity of eHSP measurements for identifying individuals who have acquired the heat acclimated phenotype.

#### Intracellular heat shock proteins

Although sample collection is slightly more technically challenging than for the measurement of eHSP, intracellular HSP70 (iHSP) content has been quantified within PBMCs in a large number of HA studies (e.g. [5, 110, 117, 122, 127, 218]), although more invasive measurements of muscle HSP content are sometimes reported [208]. Research examining iHSP levels during HA in humans has shown that shorter durations of HA, e.g. 1 to 2 days [122, 218] do not typically result in a measurable increase in iHSP70 levels, whereas significant increases in iHSP70 content may develop from 3 days onwards [110], and are commonly reported over 6 to 10 days of active HA [5, 110, 117, 122, 127, 218]) where a progressive accumulation of iHSP70 may be evident [110]. Indeed, a recent meta-analysis concluded that HA has a large significant effect overall on iHSP70 protein expression (Hedges’ *g* = 0.97), with the number of days of HA a significant moderator of the iHSP response [142]. However, it is interesting to note that Watkins et al. [208] did not detect any significant increase in muscle HSP content after completion of a 7-day active HA intervention, but the reasons for the differences between this study and those utilising the measurement of iHSP70 levels in PBMCs is not clear. Likewise, Morton et al. [138] demonstrated that passively elevating core and muscle temperature to levels encountered during aerobic exercise did not increase skeletal muscle iHSP, implying non-thermal factors elicit the elevations in muscle HSP content seen with exercise in the heat.

In addition, whilst the majority of research has focused on iHSP70/HSP72, a small number of studies have examined the effect of HA on iHSP90. First among these were McClung et al. [127] who demonstrated that the increase in iHSP90 (21.1%) was similar to the increase in iHSP72 (+ 17.7%) over a 10-day HA intervention. Similarly, Gibson et al. [66] demonstrated that iHSP90α mRNA changes over the course of HA paralleled the changes in iHSP70 mRNA, with both increasing to a similar extent during exercise-heat stress on day 1 and 10 of an HA intervention. McClung et al. [127] also reported a significant correlation (*r*^2^ = 0.89) between the percentage change in AUC for a deep body temperature of > 38.0 °C and ex vivo HSP90 inducibility between days 1 and 10 of the HA intervention, but not HSP72, suggesting that iHSP90 could have some benefit over iHSP70 in terms of identifying individuals who have acquired a given level of adaptation to heat, possibly because HSP90 plays a functionally important role in the regulation of cutaneous blood flow during exercise-heat stress [59]. However, it is also important to note that other stressors such as exercise [113] and hypoxia can result in increased iHSP, albeit possibly at a lower level than that induced by exposure to heat stress [110] and there is also some evidence for seasonal variation in iHSP levels [217].

### Sweat biomarkers

Whilst biomarker development has traditionally focused on blood and urine analysis, there is a growing realisation of the potential of sweat analysis in a biomarker context [199], driven in part by technological developments in wearable sensor technology [151]. Although sweat is primarily composed of water, it also contains a mixture of chemicals in varying concentrations, including electrolytes (Na^+^, Cl^−^, K^+^, Ca_2_^+^, Mg_2_^+^, Fe_2_^+^), micronutrients (vitamins), metabolites (e.g. glucose, lactate, ammonia, urea, bicarbonate, amino acids, ethanol), cytokines, and cortisol [13]. The recent application of high-throughput metabolomic and proteomic approaches (e.g. [82, 186]), has enabled further quantification of an extensive range of molecules in sweat, which may have future utility in a biomarker context (see Sect. “Challenges and opportunities” for further discussion of ‘omics’ approaches). However, whilst the vast majority of these molecules have yet to be examined within the context of HA, reductions in the electrolyte content of eccrine sweat, and specifically sweat sodium (Na^+^) and chloride (Cl^−^), with HA are well established [205] and may result from the increased eccrine gland responsiveness to aldosterone [96] which will increase sweat Na^+^ reabsorption by increasing the activity of Na^+^–K^+^-ATPase on the basolateral membrane in the eccrine sweat duct [13].

Recently, the changes in sweat electrolyte concentration following HA have been assessed by meta-analysis with both Na^+^ and Cl^−^ demonstrating a large effect-size for the reduction in the concentration of both sweat Na^+^ (Hedges’ g = − 0.94) and sweat Cl^−^ (Hedges’ g = − 2.02) [205]. This meta-analysis also concluded that the concentration of sweat K^+^ is unchanged with HA, a finding that is consistent with recent primary research examining the daily time course of changes in sweat electrolyte concentration over a 10-day heat acclimation programme [97], and demonstrating that the reductions in sweat Na^+^ and Cl^−^ concentration were significant from the third day of HA onwards. Klous et al. [97] also measured sweat lactate concentration over the course of the HA intervention, which broadly paralleled the time course of reductions in sweat Na^+^ and Cl^−^, becoming significant from day 6 onwards and appearing to be caused by a greater dilution due to an increased local sweat rate, rather than a reduced excretion rate [97]. The observation of a reduced sweat lactate concentration with HA has been replicated by others (e.g. [209]). Given that the changes in the concentration of the various sweat electrolytes may occur through different mechanisms, each may provide subtly different information about the adaptive process to heat. For instance, sweat Na^+^ concentration may yield information about the reabsorption rates of Na^+^ by sweat ducts, which may be linked to fluid-regulatory balance, whilst sweat lactate concentration may provide useful information about alterations in sweating rate, which is linked to maximum evaporative heat loss potential, although the latter is also influenced by metabolic stress [4].

The reduction in sweat Na^+^ concentration appears to persist with longer HA regimes > 20 days [189, 191], decay when the HA is stopped [97, 143] and be less affected by diet than plasma aldosterone concentration [2]; it can also be re-established with a period of re-acclimation [97]. Together this suggests that the concentration of certain sweat electrolytes may usefully track changes in heat acclimation state, but further research is required to verify this. However, caution is warranted due to reported substantial inter and intra-individual variability in sweat Na^+^ concentration [11]. Sweat composition is influenced by multifarious factors not related to HA, including training status, genetics/disease, exercise intensity, diet, hydration status, sweat collection method and collection site [12], each of these factors may adversely impact on the utility of sweat as a biomarker of the heat-adapted state. From a practical perspective, because of the low rate of resting eccrine sweat secretion, some form of exercise-heat stress (e.g. HAST) is typically required in order to stimulate sufficient sweat production to enable collection; as we detailed in Sect. “Introduction”, this may be neither desirable nor practical. However, a significant reduction in resting sweat Na^+^ concentration has been reported following both 6- and 23-day HA programmes using pilocarpine iontophoresis [35, 189, 191]. Similarly, new technological advances incorporating the use of microfluidics and electrochemical sensing into wearable sweat patch development may support improved resting sweat collection in the future [151, 199].

## Challenges and opportunities

Based upon the information presented in Sect. “Candidate biomarkers” it is clear that, following a period of HA, the concentration of a number of readily measurable biomolecules may be reduced during exercise-heat stress (i.e. a HAST), reflecting the reduction in thermo-physiological strain across numerous body systems (see Table 1). These biomolecules might, therefore, have potential utility as biomarkers of the heat-adapted phenotype. However, beyond negating the need for invasive deep-body temperature measurements, the practical advantage of measuring these biomolecules in this way is limited, because the considerable logistical challenges and personnel burden of administering a suitably standardised HAST remain. Moreover, the extent to which such a biomarker would provide additional information over a range of simple and minimally invasive physiological measures that are already commonly employed in a HAST, such as the assessment of whole-body sweating rate (through body mass change) or sweat Na^+^ concentration, cardiovascular strain, plasma volume changes (through fingertip capillary blood sampling) or skin temperature measurement, is questionable.

**Table 1:** Summary of candidate biomarkers for assessing the heat acclimated phenotype at rest and following a Heat Acclimation State Test (HAST)

Biomarker	Medium	Post-HA baseline change			Post-HA HAST change		
Fluid regulatory		Results			Results		
Aldosterone	Blood	↑**	↓*	↔ **		↓**	↔ **
Arginine-vasopressin	Blood	↑*		↔ **		↓**	↔ **
Copeptin	Blood			↔ **		↓*	↔ *
Renin	Blood	↑*	↓*	↔ **		↓**	↔ **
Atrial natriuretic peptide	Blood		↓*	↔ **			
Pro-Atrial natriuretic peptide	Blood			↔ *			
Brain natriuretic peptide	Blood			↔ *			
Renal stress and injury							
Creatinine	Blood			↔ **		↓**	↔ **
Neutrophil gelatinase-associated lipocalin	Urine						↔ *
Kidney injury molecule-1	Urine						↔ *
Haematological							
Haematocrit	Blood		↓**				
Haemoglobin concentration	Blood		↓**				
Haemoglobin mass		↑*	↔ *				
Plasma protein	Blood	↑**		↔ *			
Erythropoietin	Blood	↑*		↔ *			
Energy homeostasis							
Triiodothyronine (T3)	Blood		↓**	↔ *			
Thyroxine (T4)	Blood		↓**	↔ *			
Orexin	Blood		↓*				
Prostaglandin E2	Blood		↓*				
Cyclooxygenase-2	Blood		↓*				
Sympathetic–adrenomedullary axis							
Adrenaline	Blood			↔ **		↓**	↔ *
Adrenaline	Urine			↔ *			
Noradrenaline	Blood			↔ **		↓**	↔ **
Noradrenaline	Urine	↑*				↓*	
Metanephrine	Blood			↔ *		↓*	↔ *
Normetanephrine	Blood		↓*	↔ *		↓**	
Hypothalamic–pituitary–adrenal axis							
Cortisol	Blood		↓*	↔ **		↓**	↔ *
Adrenocorticotropic hormone	Blood			↔ **		↓**	↔ **
Growth hormone	Blood			↔ **		↓*	↔ **
Interleukin-6	Blood			↔ **		↓**	↔ **
Interleukin-10	Blood			↔ **		↓*	
Tumour necrosis factor α	Blood			↔ **			↔ **
C-reactive protein	Blood			↔ *			↔ *
Heat shock							
Intracellular heat shock protein 70	PBMC	↑**					
Intracellular heat shock protein 70	Muscle			↔ *			
Extracellular heat shock protein 70	Blood		↓**	↔ **		↓**	↔ **
Intracellular heat shock protein 90	PBMC	↑*					
Sudomotor							
Sodium	Sweat		↓**			↓**	
Chloride	Sweat					↓**	
Potassium	Sweat						↔ **
Lactate	Sweat					↓**	

↑ = increase in concentration or level of biomolecule; ↓ = decrease in concentration or level of biomolecule; ↔  = no change in concentration or level biomolecule

*HA* heat acclimation/acclimatisation, *PBMC* peripheral blood mononuclear cell

*Study supporting identified change when heat adapted

**Multiple studies supporting identified change when heat adapted

Among the various biomolecules that we have reviewed, there is some evidence that the plasma, serum, sweat or intracellular concentration of a small subset of these may be altered in the resting state following HA (see Table 1). For the reasons outlined above, a change in the resting concentration of a given biomolecule is likely to be of greater practical relevance in the context of developing a molecular biomarker of the heat-adapted state than changes observed during a HAST. However, as we have highlighted, for many of these putative molecular biomarkers the data are limited at present to a small number of studies, or are not conclusive and further research is needed to confirm the robustness of reported baseline changes; in the majority of cases relevant data are lacking from female volunteers. Moreover, given the pronounced hypervolemia that occurs with HA, it is interesting that some of the studies reviewed have not reported appropriate adjustment of biomarker concentrations to account alterations in plasma volume [124]. Without these adjustments, for a fixed circulating amount of a molecule the resting plasma/serum concentration will typically be reduced following a period of HA, but as a consequence of the hypervolemia rather than a change in the amount of the circulating molecule. Differences in the application of this adjustment among the studies that we have reviewed may have contributed to some of the inter-study variability.

Moreover, HA has been described as a continuum of many parallel processes [120]. For example, fluid regulatory changes and the associated haematological effects may be observed very quickly (e.g. 2–3 days; [14]), whereas cytoprotective (> 3 days; [142]) and sudomotor adaptions (e.g. 7–14 days; [175]) typically develop over longer timescales. Thus, whilst the measurement of a given biomolecule may provide useful information about changes in a particular biological system, it may not necessarily provide information about the integrated adaptive process to heat, i.e. the overall state of ‘heat readiness’. For instance, a change in a biomarker indicative of expanded plasma volume (e.g. haemoglobin concentration) might precede a change in a biomarker of cytoprotection (e.g. iHSP70) or sudomotion (e.g. sweat Na^+^ concentration). Temporal dissociation between changes in the concentration of some of these putative biomarkers and the induction of the ‘hallmark’ indices of the heat-adapted state may confound the development of a biomarker of the heat-adapted state. Allied to this, we are not aware of any research that has examined the relationship between the rate of decay in hallmark indices of HA that occurs once the heat stressor is removed and the kinetics of many of these biomolecules, but it is known that, similar to the induction time-course, the decay rate of the various hallmark indices of heat adaption is also variable [32]. Therefore, research is needed to better understand the temporal relationship between changes in biomolecule concentration and the “hallmark” indices of the heat-adapted phenotype during, and following, an HA programme.

From the information presented in Sect. “Candidate biomarkers”, it is also apparent that the concentration of many of these putative biomarkers has the potential to be altered by a variety of non-thermal stressors. This is perhaps unsurprising given the multi-systemic nature of the adaptions to heat, but will likely result in commonalities in the biochemical changes induced by heat exposure and those occurring with exposure to other stressors such as: exercise; infection; dietary change (calorific intake or composition); disease; psychological stress; other environmental stressors such as hypoxia. Such an overlap is likely to adversely impact on the sensitivity and specificity of a given biomolecule in providing useful information about the heat-adapted phenotype. However, it has been recognised that exposure to different stressors may invoke common adaptive pathways (e.g. heat and hypoxia), and that the adaptions occurring through exposure to one stressor may confer both acclimatory (e.g. whole body) and cytoprotective (e.g. cellular) effects when exposed to another stressor [40]. As such, it is unclear to what extent, if any, the potential overlap between changes in biomolecules occurring with exposure to non-thermal stressors would present a meaningful problem, given that such changes may still be indicative of an improved ability to tolerate heat stress. Similarly, whilst seasonal changes may occur in a number of the biomolecules that we have identified, these may simply be reflective of natural acclimatisation to an increased ambient temperature, and would therefore be expected to improve tolerance to thermal stress.

Taken together, the evidence presented in this review suggests that, at present, no single biomolecule (i.e. a univariable approach) will provide sufficient information or demonstrate adequate sensitivity and specificity to act as a biomarker of the heat-adapted phenotype. However, the use of multivariable modelling approaches has been successful employed in other fields to develop more powerful biomarkers with superior diagnostic ability (e.g. [48]). Such an approach may be particularly well suited to the multi-system adaptions that are characteristic of HA. Ensuring that such a model includes variables indicative of a range of body systems associated with HA, as well as potentially including information from other factors known to influence thermoregulation such as anthropometric (e.g. body mass, body composition, body surface area) and demographic factors (e.g. age and gender) [77]), might overcome some of the challenges identified.

Moving beyond multivariable approaches incorporating small numbers of variables (e.g. < 10 variables), the future application of ‘omics’ approaches including metabolomics and transcriptomics may prove fruitful. The metabolome is directly linked to changes in cell metabolism that are caused by environmental stimuli, as well as diseases, and utilises the measurement of hundreds to thousands of metabolites within a biological medium. Whilst this approach has already proved fruitful in biomarker development [98], it is not without challenges including strict technical requirements related to sample storage and analysis, high inter-individual variability, the potential confounding effects of a range of demographic factors, and the need for large samples sizes (e.g. *n* = 200–400) to protect against false discovery rate [203]. The ‘n’ for most HA studies is an order of magnitude lower than that recommended for metabolomics studies owing to the time and resource intensive nature of this type of work [205].

The transcriptome is the complete set of all RNAs transcribed by certain tissues or cells and has emerged as a powerful approach for investigating the molecular response to environmental stressors [220]. Indeed, the transcriptomic response of human PBMCs to heat has recently been reported, and is characterised by significant gene suppression and differentially expressed genes encoding proteins involved in proteostasis, energy metabolism, cell growth and proliferation, and cell death and survival [18]. However, the significance of these observations in terms of developing a biomarker of heat adaptation is, as yet, unclear. Finally, technological advances in the development of sensors for non-invasive and minimally invasive continuous analyte monitoring are now enabling the development of wearable devices that are overcoming historical challenges for biological sample analysis [60], and in combination with real time biomonitoring techniques and more complex analysis approaches (e.g. the use of resting heart rate variability; [49]), may pave the way for addressing the aforementioned temporal problems.

## Conclusions

Quantifying the level of phenotypic adaption to heat from the measurement of a biomolecule(s) may be desirable in a number of contexts (e.g. defence, occupational, sporting), particularly with emerging technological developments. Such a biomarker may have utility to characterise the ‘heat readiness’ of an individual, thereby reducing heat-illness risk and increasing operational and training capacity. This narrative review of literature has shown that the concentration of a number of biomolecules implicated in fluid regulation, energy homeostasis, sympatho-adrenal balance, inflammation, sudomotor adaption and acquired thermal tolerance may be altered when an individual is exposed to a standard heat stress following a period of HA. However, only a small subset of these biomolecules appears to be altered at baseline. Moreover, for many of these putative biomarkers the data are equivocal and/or limited at present to a small number of studies and (male) participants. Given the multi-systemic nature of adaption to heat, we speculate that no single biomolecule is likely to have sufficient sensitivity and specificity as a biomarker of the heat-adapted phenotype when used in isolation. Thus, we propose that future research should verify the robustness of the change in the baseline concentration of the identified biomolecules following a period of HA and their temporal association with different ‘hallmark indices’ of the heat-adapted phenotype (i.e. physiological biomarkers, including appropriate deep body temperate measurements). The use of multivariable approaches that include biomarkers providing information about a range of physiological systems should also be explored.

## Data Availability

Data sharing is not applicable to this article as no datasets were generated or analysed during the current study.
